# Machine learning for predicting metabolic-associated fatty liver disease including NHHR: a cross-sectional NHANES study

**DOI:** 10.1371/journal.pone.0319851

**Published:** 2025-03-18

**Authors:** Liyu Lin, Yirui Xie, Zhuangteng Lin, Cuiyan Lin, Yichun Yang

**Affiliations:** 1 The First Clinical Medical College of Guangzhou University of Chinese Medicine, Guangzhou, China,; 2 The First Affiliated Hospital of Guangzhou University of Chinese Medicine, Guangzhou, China,; 3 Guangzhou Red Cross Hospital, Guangzhou, China; The First Affiliated Hospital of Wenzhou Medical University, CHINA

## Abstract

**Objective:**

Metabolic - associated fatty liver disease (MAFLD) is a common hepatic disorder with increasing prevalence, and early detection remains inadequately achieved. This study aims to explore the relationship between the non-high-density lipoprotein cholesterol to high-density lipoprotein cholesterol ratio (NHHR) and MAFLD, and to establish a predictive model for MAFLD using NHHR as a key variable.

**Methods:**

All participants were selected from the NHANES cohort, spanning from 2017 to March 2020. Multiple linear regression models were employed to examine the relationship between the non-high-density lipoprotein cholesterol to high-density lipoprotein cholesterol ratio (NHHR) and the controlled attenuation parameter (CAP). To explore the non-linear association between NHHR and CAP, smooth curve fitting and restricted cubic splines (RCS) of the adjusted variables were utilized. Subgroup analyses were conducted to identify variations in the relationships between the independent and dependent variables across different populations. Finally, a metabolic - associated fatty liver disease (MAFLD) prediction model was developed using seven machine learning methods, including eXtreme Gradient Boosting (XGBoost), Light Gradient Boosting Machine (LightGBM), Multilayer Perceptron (MLP), Random Forest, Support Vector Machine (SVM), K-Nearest Neighbors (KNN), and logistic regression. The SHAP (SHapley Additive exPlanations) value was employed to interpret the importance of various features.

**Result:**

Weighted multiple linear regression models revealed a significant positive correlation between the NHHR and the CAP (Beta =  7.42, 95% CI: 5.35-9.50, P <  0.001). Smooth curve fitting and RCS demonstrated a non-linear relationship between NHHR and CAP. Subgroup analyses indicated that this relationship was more pronounced in females. Among the seven machine learning predictive models incorporating NHHR, the XGBoost algorithm exhibited the highest predictive performance, with an area under the curve (AUC) of 0.828. Furthermore, NHHR was identified as the second most important feature in the SHAP analysis, following body mass index (BMI), highlighting its potential in predicting MAFLD.

**Conclusion:**

A significant positive correlation was identified between the NHHR and the CAP. The inclusion of NHHR in the XGBoost predictive model for MAFLD demonstrated robust predictive capability, providing a valuable tool for the early detection of MAFLD with considerable clinical application potential.

## 1 Introduction

The increasing prevalence of metabolic - associated fatty liver disease (MAFLD) and its advanced stage, non-alcoholic steatohepatitis (NASH), poses a significant public health challenge worldwide, paralleling the global rise in obesity and metabolic syndrome [1]. MAFLD, characterized by excessive fat accumulation in the liver not attributed to alcohol consumption, can progress to NASH, fibrosis, cirrhosis, and even hepatocellular carcinoma, making early detection and intervention crucial [2,3].

The non-High-Density Lipoprotein Cholesterol (non-HDL-C) to High-Density Lipoprotein Cholesterol (HDL-C) ratio (NHHR) has gradually emerged as a novel composite index for assessing lipid profiles implicated in the pathogenesis of atherosclerosis [4]. In a consensus report by the American Diabetes Association and the American College of Cardiology in 2008, it was found that non-HDL-C was more adept at identifying individuals at high risk for cardiovascular disease (CVD) than low-density lipoprotein cholesterol (LDL-C) [5]. Orakzai et al. conducted an observational study involving 1611 participants and observed a significant increase in the prevalence of coronary artery calcification among those in the highest quartile of non-HDL-C levels (P < 0.01) [6]. Building upon these findings, the NHHR index, reflective of non-HDL-C metabolism, has gradually supplanted singular biomarkers, becoming a crucial predictor of cardiovascular and other lipid metabolism disorders. On the other hand, HDL-C plays a protective role in the onset of MAFLD. Recent years have witnessed a growing interest in Mendelian Randomization (MR) studies, which leverage genetic variants from genome-wide association studies (GWAS) data to explore causal relationships. This novel approach has provided additional layers of evidence. For instance, an analysis of 35 modifiable risk factors in relation to MAFLD identified that increased levels of HDL-C could decrease the risk of MAFLD [7]. Similarly, another MR study investigating the impact of lifestyle and metabolic factors on MAFLD found that higher levels of HDL-C were associated with a reduced incidence of MAFLD (OR = 0.84) [8].

The intricate relationship between non-HDL-C levels and MAFLD, as well as the progression to liver fibrosis, has garnered significant attention, underpinned by burgeoning evidence that underscores the pathophysiological linkages connecting lipid metabolism aberrations to hepatic fat accumulation and fibrogenesis [5,9,10].

The application of machine learning algorithms has brought new breakthroughs and development opportunities for disease prediction models in recent years. Among them, various classical machine learning algorithms have demonstrated significant application potential. As prominent representatives of gradient boosting algorithms, XGBoost and LightGBM have exhibited excellent performance in handling large-scale data and deciphering complex feature relationships. XGBoost leverages second-order Taylor expansion and regularization techniques to effectively prevent model overfitting and ensure model stability and generalization capability. LightGBM, on the other hand, significantly improves computational efficiency and reduces resource consumption through innovative histogram-based algorithms. As a neural network model, Multilayer Perceptron (MLP) possesses powerful learning capabilities, which can effectively capture complex nonlinear relationships in data, providing insights into the underlying complex mechanisms of diseases. Random Forest, based on an ensemble learning approach of decision trees, demonstrates robust performance, being able to withstand noise and outliers in data, as well as providing effective assessment of feature importance to help researchers screen for key influential factors. Support Vector Machine (SVM) has shown outstanding performance in small-sample and nonlinear classification tasks, capable of accurately separating data of different classes. The K-Nearest Neighbors (KNN) algorithm is simple and intuitive, based on instance learning, and can quickly provide effective prediction results in many scenarios. Logistic regression has the advantages of being easy to understand and interpret, with model coefficients intuitively reflecting the direction and degree of influence of each factor on disease occurrence. Given that the data in this study is mainly structured clinical indicators, which do not fit the data patterns that deep learning models excel at, such as images, text, or time series data, and also have relatively poorer model interpretability, this study prioritizes the use of traditional machine learning algorithms to build models. The goal is to quickly obtain valuable information and prediction results, laying a solid foundation for potential future deep learning research.

Despite mounting evidence linking non-HDL-C to the occurrence and progression of MAFLD, the relationship between the NHHR index and MAFLD has not been thoroughly explored in previous studies. In this study, data spanning from January 2017 to March 2020 were meticulously analyzed, utilizing the comprehensive NHANES dataset to examine the association between the NHHR index and non-alcoholic steatosis. By employing machine learning techniques, a predictive model was constructed with the objective of elucidating the prevalence and correlates of MAFLD among U.S. adults. This approach has provided a practical tool for the early detection and monitoring of liver disease progression, thereby enhancing the understanding of its epidemiology and underlying mechanisms. The integration of advanced analytical methods has enabled a more nuanced exploration of the complex interplay between liver health indicators, offering significant insights into the effective management and prevention strategies for liver-related disorders.

## 2 Methods

### Data source

The NHANES program, administered by the National Center for Health Statistics (NCHS), aimed to assess the health and nutritional status of the U.S. population through information gathered via questionnaire surveys, physical examinations, and laboratory tests. NHANES employed a complex, multi-stage sampling design to obtain nationally representative samples of approximately 5,000 individuals annually, with the database being updated every two years. The NHANES survey protocol was approved by the NCHS Institutional Review Board, and informed written consent was obtained from all participants. Additionally, all information in the database was made available to the public (https://wwwn.cdc.gov/nchs/nhanes/Default.aspx), thereby exempting our study from further ethical review [11].

### Study population

Since 2017, the NHANES working group has been engaged in the collection of FibroScan^®^ measurements from participants, utilizing ultrasound and Vibration-Controlled Transient Elastography (VCTE) to obtain liver stiffness measurements (LSM). Concurrent measurements of ultrasound attenuation related to hepatic steatosis were taken, and the controlled attenuation parameter (CAP) was documented as an indicator of liver fat content. Due to the COVID-19 pandemic, the NHANES survey was temporarily halted in March 2020, thus our study period spans from January 2017 to March 2020 [12].

**Fig 1:**
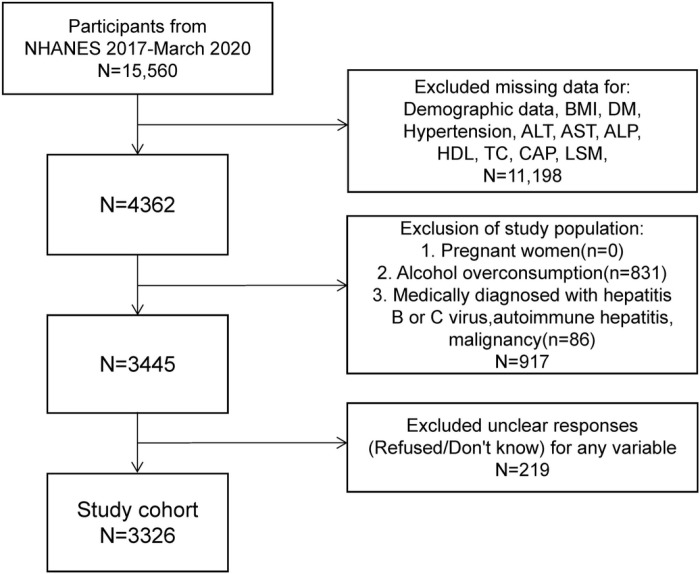
Characteristics of participants. NHHR, non-high-density lipoprotein cholesterol to high-density lipoprotein cholesterol ratio.

Data for this study were extracted from the NHANES database for the 2017-2020 cycle. The study included adults aged ≥18 years with available FibroScan^®^ measurements. Participants who were pregnant, had a history of excessive alcohol consumption (defined as an average of 15 drinks or more per drinking day, or consuming alcohol 3 to 4 times per week or more), hepatitis B or C virus, autoimmune liver diseases, or a history of malignancy were excluded. (Fig 1)

### Assessment of NHHR

The data for NHHR calculation were derived from laboratory data within NHANES, specifically extracted from the files “P_HDL.xpt” and “P_BIOPRO.xpt” to obtain HDL (mg/dl) and TC (mg/dl) data, respectively. The NHHR was computed using the formula proposed in prior studies, involving subtracting HDL from total cholesterol to obtain non-HDL-C, and subsequently dividing it by HDL [13].

### Covariates

To more rigorously ascertain the precise and independent effect of NHHR on Liver Fibrosis and MAFLD, potential confounders that could obscure the relationship were incorporated into a multivariable adjustment model. This model included variables such as gender, age, ethnicity, educational attainment, income relative to the poverty index (PIR), body mass index (BMI), diabetes, hypertension, and levels of alanine aminotransferase (ALT) (U/L), aspartate aminotransferase (AST) (U/L), and alkaline phosphatase (ALP) (U/L). These factors were selected for their potential to introduce confounding effects into the analysis, thereby allowing for a more nuanced and accurate assessment of NHHR’s independent impact on Liver Fibrosis and MAFLD.

The PIR represents the ratio of household income to poverty, which is calculated using the poverty guidelines of the Department of Health and Human Services (HHS) as a measure. Based on previous studies [14], PIR was categorized as below 1.3, 1 to 3, and above 1.8. BMI was categorized as below 25 kg/m^2^, 25 to 30 kg/m^2^, and above 30 kg/m^2^, representing normal weight, overweight, and obesity, respectively. Considering that hypertension can lead to an increase in portal venous system pressure, exacerbating the progression of liver fibrosis [15], and insulin resistance is a hallmark of type 2 diabetes, which promotes hepatic fat accumulation, inflammation, and fibrosis, closely associated with the occurrence and progression of hepatic steatosis [16], we included hypertension and diabetes in the model. Participants with missing or unclear responses (Refused/Don’t know) for any variable were excluded.

### Measurement criteria for MAFLD

The diagnosis of MAFLD follows the criteria outlined in the international expert consensus statement published in 2020 [17]. MAFLD is diagnosed when hepatic steatosis and metabolic dysfunction are present simultaneously.

The degree of hepatic steatosis was assessed using CAP. Currently, no precise threshold exists to clearly define the presence or absence of hepatic steatosis (S0/S1). However, based on widely accepted standards, a CAP value below 238 dB/m is considered indicative of the absence of hepatic steatosis, whereas a value exceeding 238 dB/m suggests the presence of hepatic steatosis [18–21].

Metabolic dysfunction manifests in various forms, with parameters such as BMI being influential. In this study, the commonly used classification method was employed: a BMI of less than 25 kg/m² was classified as normal weight, while a BMI greater than 25 kg/m² was classified as overweight, in accordance with the consensus statement. Furthermore, diabetes and hypertension, which are typical components of metabolic dysfunction, were also considered as indicators of metabolic dysfunction in our diagnostic criteria.

Finally, in accordance with the diagnostic requirements of the consensus statement, other potential causes of liver-related issues were excluded. In this study, participants with a history of excessive alcohol consumption, those infected with hepatitis B or C viruses, individuals with autoimmune liver diseases, and patients with a history of malignant tumors were excluded.

In summary, MAFLD is diagnosed when the CAP value is ≥238 dB/m and at least one of the following conditions is present: diabetes, hypertension, or a BMI ≥  25 kg/m².

### Statistical analysis

All Statistical analyses were performed using R version 4.2.3 and python version 3.11.4. Weighted samples, stratification, and clustering from the NHANES database were employed to produce nationally representative estimates, following analytical guidelines provided by the National Center for Health Statistics. Continuous variables were represented as weighted means and standard errors, while categorical variables were reported as unweighted counts and weighted proportions [22].

Multivariate linear regression was utilized to examine the independent associations between NHHR and LSM or CAP across three distinct models. Model 1 did not include adjustments for covariates. Model 2 incorporated adjustments for sex, age, race, PIR, and educational attainment. Model 3 extended Model 2 by including adjustments for BMI, hypertension, diabetes, ALT, AST, and ALP. A two-sided p-value less than 0.05 was considered significant.

### Construction of machine learning models

In Model 3 (continuous), the variables significantly associated with the CAP (P <  0.05) were used as independent variables, while the measured CAP was treated as the dependent variable. The diagnosis of MAFLD was determined based on established measurement criteria. The study population was randomly sampled, with a random seed set to 10. A test set consisting of N =  998 cases (30.00%) was randomly selected from the overall sample, while the remaining samples were utilized for the training set to perform 2-fold cross-validation. Diagnostic models for MAFLD were constructed using seven machine learning methods, including eXtreme Gradient Boosting (XGBoost), Light Gradient Boosting Machine (LightGBM), Multilayer Perceptron (MLP), Random Forest, Support Vector Machine (SVM), K-Nearest Neighbors (KNN), and logistic regression. The predictive performance of each model was assessed by calculating the area under the curve (AUC) value.

SHAP (SHapley Additive exPlanations) values, grounded in cooperative game theory, are employed to interpret the significance of various features within machine learning models. By quantifying the contribution of each variable to the model’s predictions, this method enhances model transparency. It elucidates how input features influence outputs, thereby facilitating a deeper understanding of the underlying relationships within the data. In this study, SHAP values are utilized to assess the importance of individual features in the predictive model.

To better interpret the model locally and validate its reliability, we employed a random sampling method to select samples of sizes 200, 500, and 1000. This approach was used to assess the model’s predictive performance and the importance of individual features.

### Ethics approval

The NCHS Research Ethics Review Board approved the NHANES survey protocol. All participants provided written informed consent. Additionally, as all information in the NHANES database is available to the public, our study was exempt from ethical review.

## 3 Results

### Characteristics of participant

A total of 3,326 participants were included in this study (Table 1), with a mean age of 46.6 ±  16.2 years, of whom 46.6% (1,549 individuals) were male. The mean NHHR level was 2.56. Participants were categorized into four groups (Q1 to Q4) based on the quartiles of NHHR: Q1 (0.28-1.87), Q2 (1.87-2.56), Q3 (2.56-3.45), and Q4 (3.45-17.74). The complete data of the study population included can be found in S1 Table.

**Table 1.** Characteristics of the study population. NHHR: non-high-density lipoprotein cholesterol to high-density lipoprotein cholesterol ratio, PIR: poverty income ratio, CAP: controlled attenuation parameter.

Characteristic	NHHR				
	Q1 (N = 832)	Q2 (N = 831)	Q3 (N = 831)	Q4 (N = 832)	P-value
Age (years)	43.0 (40.5-45.5)	44.2 (42.1-46.3)	47.1 (44.2-50.0)	45.4 (43.9-47.0)	0.03
Gender					<0.001
Female	569 (72.4%)	500 (62.1%)	414 (47.1%)	294 (35.3%)	
Male	263 (27.6%)	331 (37.9%)	417 (52.9%)	538 (64.7%)	
Race					<0.001
Non-Hispanic white	280 (65.0%)	317 (66.8%)	302 (63.7%)	321 (65.2%)	
Non-Hispanic black	261 (13.5%)	222 (11.3%)	193 (9.6%)	126 (5.9%)	
Mexican American	92 (7.4%)	90 (7.3%)	119 (10.1%)	142 (11.5%)	
Other Hispanic	199 (14.1%)	202 (14.6%)	217 (16.6%)	243 (17.3%)	
PIR					0.059
<1	151 (10.6%)	114 (9.5%)	132 (11.8%)	143 (11.4%)	
1-3	340 (34.9%)	325 (27.9%)	362 (35.2%)	360 (32.4%)	
>3	341 (54.5%)	392 (62.6%)	337 (53.1%)	329 (56.2%)	
Education level					0.002
No higher education received	275 (28.8%)	251 (28.1%)	319 (36.8%)	358 (38.9%)	
Higher education or above	557 (71.2%)	580 (71.9%)	512 (63.2%)	474 (61.1%)	
BMI					<0.001
<25	372 (50.4%)	215 (28.9%)	133 (14.9%)	76 (8.1%)	
25-30	230 (24.6%)	239 (28.6%)	269 (34.1%)	268 (31.2%)	
>30	230 (24.9%)	377 (42.5%)	429 (51.0%)	488 (60.6%)	
Diabetes					0.067
Yes	100 (8.3%)	100 (7.5%)	104 (11.3%)	128 (12.7%)	
No	732 (91.7%)	731 (92.5%)	727 (88.7%)	704 (87.3%)	
Hypertension					<0.001
Yes	247 (21.1%)	265 (26.1%)	286 (32.4%)	303 (34.1%)	
No	585 (78.9%)	566 (73.9%)	545 (67.6%)	529 (65.9%)	
ALT (U/L)	18.1 (16.9-19.4)	20.7 (18.8-22.5)	22.7 (21.4-24.0)	27.6 (25.9-29.3)	<0.001
AST (U/L)	20.5 (19.4-21.5)	20.7 (19.6-21.7)	20.5 (19.7-21.3)	22.1 (21.2-23.0)	0.028
ALP (U/L)	67.5 (65.4-69.6)	73.3 (70.8-75.9)	75.7 (73.3-78.1)	80.1 (77.1-83.1)	<0.001
HDL (mg/dL)	67.7 (66.0-69.3)	56.3 (55.3-57.3)	47.8 (47.0-48.6)	39.7 (38.8-40.6)	<0.001
TC (mg/dL)	162.2 (157.9-166.5)	179.9 (176.8-183.1)	189.3 (185.1-193.5)	214.8 (210.1-219.6)	<0.001
CAP	229.5 (223.7, 235.2)	251.7 (246.2, 257.3)	274.5 (270.0, 279.0)	294.9 (288.4, 301.4)	<0.001

Compared to participants in Q1, those in Q4 had a higher proportion of males, as well as higher proportions of BMI, DM, and hypertension. Furthermore, levels of ALT, AST, ALP, LSM, and CAP were significantly elevated among Q4 participants. Interestingly, we observed a gradual decrease in the proportion of Non-Hispanic Black individuals as the NHHR index increased, suggesting that black individuals may be less prone to non-HDL cholesterol accumulation compared to other ethnic groups.

### The association between NHHR and CAP

Utilizing the same covariate-adjusted models, we further investigated the relationship between NHHR and CAP. The multivariate linear analysis results assessing the association between NHHR and CAP are presented in Table 2. When treated as a continuous variable, NHHR demonstrated a significant positive correlation with CAP, maintaining statistical significance after adjusting for all variables (Beta = 7.42, 95% CI: 5.35-9.50, P <  0.01). This positive association persisted even when NHHR was categorized into quartiles.

**Table 2.** The association between NHHR and CAP.

NHHR	Model 1		Model 2		Model 3	
	Beta (95%CI)	p-value	Beta (95%CI)	p-value	Beta (95%CI)	p-value
Continuous	18.35 (16.12, 20.57)	<0.001	16.50 (14.05, 18.96)	<0.001	7.42 (5.35, 9.50)	<0.001
Quantiles						
Q1	Reference		Reference		Reference	
Q2	22.28 (15.16, 29.40)	<0.001	20.41 (13.21, 27.60)	<0.001	5.82 (-1.44, 13.08)	0.160
Q3	45.01 (37.48, 52.54)	<0.001	39.17 (32.44, 45.90)	<0.001	15.67 (9.18, 22.16)	0.002
Q4	65.42 (58.93, 71.92)	<0.001	59.39 (52.15, 66.63)	<0.001	25.53 (17.16, 33.89)	<0.001

**Fig 2:**
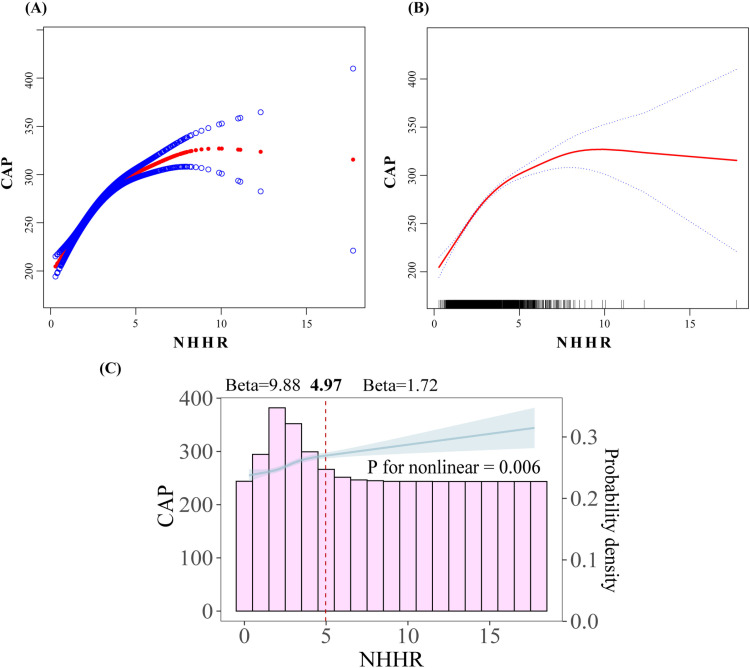
Smooth curve fitting results for NHHR and CAP. (A) Scatter plot of NHHR against CAP distribution. (B) Fitted curve of NHHR against CAP. (C) RCS curve after adjusting all variables.

To validate our findings and explore potential non-linear relationships, a non-weighted smooth curve fitting was first employed to illustrate the variation between NHHR and CAP, as shown in Fig 2A and 2B. The graphical analysis revealed a positive trajectory of CAP as the NHHR index increased. Notably, a potential decline in CAP was observed when NHHR exceeded 10. However, due to the scarcity of individuals with NHHR values above 10 and the broad 95% confidence intervals for CAP at these high values, the observed negative growth effect was deemed unreliable. Consequently, we further applied a restricted cubic splines (RCS) model, adjusting for NHANES sampling weights and all relevant covariates. The results confirmed the existence of a non-linear relationship between NHHR and CAP (P for nonlinearity =  0.006). However, no evidence was found to support the hypothesis that CAP declines as NHHR increases.

Specifically, a significant change in effect size occurred at NHHR =  4.97. When NHHR was below 4.97, the beta value was 9.88, indicating a rapid increase in CAP. In contrast, when NHHR reached or exceeded 4.97, the beta value decreased to 1.72, indicating a slower rate of increase. Overall, CAP continued to rise with increasing NHHR values, as illustrated in Fig 2C.

### Subgroup analysis of correlation between NHHR and CAP

**Fig 3:**
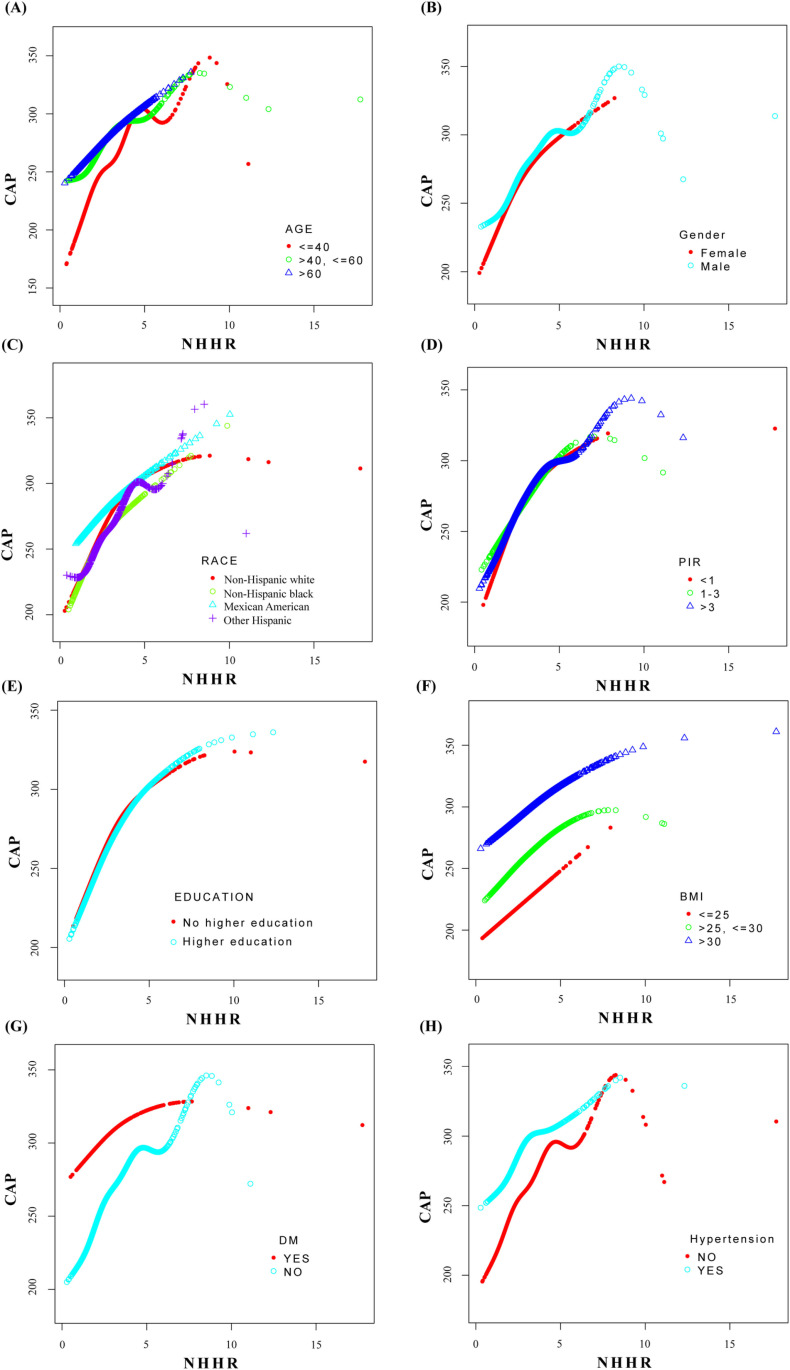
Smooth curve fitting in subgroup. The variations in NHHR and CAP were sequentially displayed across subgroups divided by gender, age, race, PIR, educational level, BMI, diabetes, and hypertension, different colored curves were employed to represent distinct groups within the population.

To validate the stability of this positive correlation, subgroup analyses were conducted across variables including gender, age, race, PIR, educational level, BMI, diabetes, and hypertension to assess variations of this relationship across different populations. In the fully adjusted model, the subgroup analyses revealed that, compared to males, the association was significantly stronger in females (Beta = 9.06, 95% CI: 6.60-11.52, P < 0.001). On the other hand, this relationship was not statistically significant in Mexican Americans (P = 0.051), individuals with a BMI < 25 (P = 0.146), and diabetic patients (P = 0.117). However, the P-values for interaction among these three groups were not significant (P > 0.05), indicating no interaction effect between race, BMI, and diabetes in the relationship between NHHR and CAP. The results of the subgroup analyses are presented in Table 3. In order to provide a visual representation of the relationship between the two variables in different subgroups, a stratified smoothed fitting curve analysis was conducted, as illustrated in Fig 3.

**Table 3.** Results of subgroup analysis.

Characteristic	N	Beta (95%CI)	p-value	P for interaction
Age				0.751
< 40	1347	7.86 (3.88,11.84)	0.008	
40-60	1172	6.36 (3.47,9.25)	0.005	
>60	807	8.18 (3.29,13.06)	0.017	
Gender				0.14
Female	1777	9.06 (6.60,11.52)	<0.001	
Male	1549	6.08 (3.26,8.89)	0.004	
Race				0.395
Non-Hispanic white	1220	7.68 (4.78,10.57)	0.004	
Non-Hispanic black	802	7.62 (5.06,10.19)	0.002	
Mexican American	443	4.26 (0.99,7.52)	0.051	
Other Hispanic	861	7.49 (4.78,10.19)	0.003	
PIR				0.685
<1	828	7.00 (4.08,9.92)	0.003	
1-3	405	5.57 (1.85,9.29)	0.026	
>3	2093	7.53 (4.79,10.27)	0.002	
Education level				0.778
No higher education received	1203	7.53 (5.01,10.05)	<0.001	
Higher education or above	2123	7.08 (4.43,9.72)	0.001	
BMI				0.274
<25	796	3.40 (-0.59,7.38)	0.146	
25-30	1006	7.87 (5.32,10.43)	<0.001	
>30	1524	7.72 (4.58,10.86)	0.003	
Diabetes				0.094
Yes	432	3.34 (-0.32,7.01)	0.117	
No	2894	8.23 (5.61,10.85)	<0.001	
Hypertension				0.214
Yes	2225	6.06 (3.71,8.42)	0.002	
No	1101	9.73 (5.23,14.24)	0.004	

### Predictive model for MAFLD

**Fig 4:**
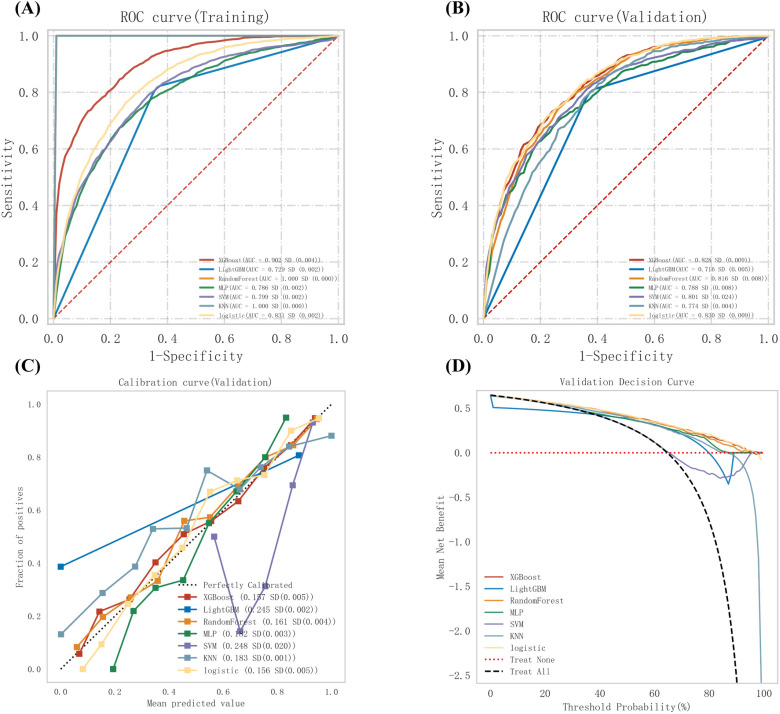
ROC Curves for training and validation sets (A, B), and calibration and decision curves (C, D) of six machine learning lodels for predictive performance. XGBoost, eXtreme Gradient Boosting; LightGBM, Light Gradient Boosting Machine; MLP, Multilayer Perceptron; SVM, Support Vector Machine; KNN, K-Nearest Neighbors.

A variety of machine learning algorithms were employed for the classification task, with the performance of each model summarized as follows: The Random Forest model exhibited the best performance in the training set (AUC =  1.000), while the Logistic Regression model demonstrated the highest performance in the validation set (AUC =  0.830). The XGBoost model performed well in both the training set (AUC =  0.902) and the validation set (AUC =  0.828). It is suggested that the Random Forest model may be prone to overfitting, whereas the XGBoost model displays relatively good stability (Fig 4). Considering both model performance and stability, the XGBoost model was selected as the preferred classification model for further research.

The calibration curve and validation decision curve for the validation set present the performance of various machine learning models, further demonstrating the reasons for XGBoost being selected as the final model. The calibration curve shows that XGBoost possesses a certain degree of stability and accuracy, with a standard deviation of 0.17 and SD (0.005), being comparable to better-performing models. Although not the top in calibration, it maintains a good balance between performance and complexity. The validation decision curve reveals that XGBoost offers a significant net benefit within a certain threshold range, outperforming some models and showing potential in guiding treatment decisions when compared to the “Treat All” and “Treat None” baselines. The selection of XGBoost is based on its performance on the validation set and its suitability for the research problem. However, further analysis and validation are needed to understand its advantages and limitations in different scenarios.

**Fig 5:**
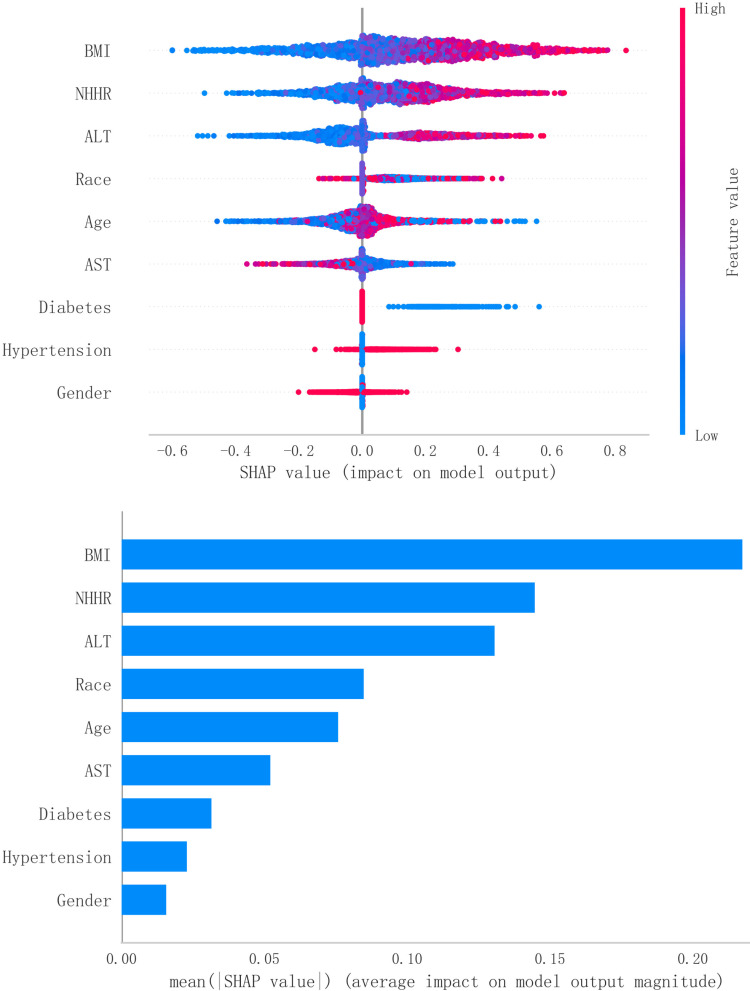
Feature importance in XGBoost model for MAFLD prediction, interpreted using **SHAP** **method.** SHAP, shapley additive explanations.

Fig 5 analyzes the importance of various features in the XGBoost model, as interpreted using the SHAP method. Through the application of SHAP values, BMI, NHHR, and ALT were identified as the top three most influential features for predicting MAFLD. The findings indicate that NHHR plays a significant role as a key predictive factor for MAFLD.

In the randomly selected cases, the validation set AUC values for sample sizes of 200, 500, and 1000 were 0.806, 0.826, and 0.747, respectively (S2-4 Table). Although there is some fluctuation in the AUC values, the model still maintains good performance. In the SHAP value-based local model interpretation, NHHR consistently ranks among the top two features. The specific feature distributions can be found in Fig S1-6.

## 4 Discussion

In this study, we utilized nationally representative data to explore the relationship between the NHHR and CAP, an indicator of fatty liver severity. After adjusting for relevant covariates, it was observed that elevated NHHR levels are positively correlated with an increased CAP, suggesting a robust association between NHHR and liver fat content. Furthermore, a clinical prediction model was developed to estimate the occurrence of MAFLD. This novel tool holds promise for early detection and management, thus underscoring NHHR’s potential as a significant marker in clinical assessments of MAFLD.

Traditionally, non-HDL-C has been recognized as a major risk factor in cardiovascular disease management. However, emerging literature, dating back to 2012, has emphasized a significant association between non-HDL-C levels and MAFLD [23,24]. In 2018, two independent studies conducted in the U.S. and China identified NHHR as a more effective predictor of MAFLD, demonstrating a stronger correlation with the disease compared to non-HDL-C alone [24,25]. By 2020, several studies had highlighted NHHR as a key independent predictor of MAFLD [26,27], reinforcing the clinical relevance of NHHR as a diagnostic tool. Beyond its application to lipid metabolism disorders, NHHR has gained attention for its association with a range of non-lipid metabolic diseases, such as depression, periodontal disease, suicidal ideation, diabetes, and abdominal aortic aneurysms [28–32]. Bart et al.’s study further demonstrated that changes in lipid profiles do not significantly impact liver fibrosis progression in hepatitis C patients, indirectly supporting our findings on the limited role of traditional lipid markers in predicting liver disease severity [33]. Thus, the use of NHHR, as opposed to conventional lipid parameters, may provide a more comprehensive understanding of the balance between atherogenic and anti-atherogenic lipoproteins, offering a novel approach for both cardiovascular risk and MAFLD severity assessments.

Our research builds upon previous work, conducting a similar yet in-depth exploration and employing statistical analysis to discern the developmental patterns of MAFLD prevalence through groupings and stratifications of MAFLD patients. Multiple data models were established for assessment and comparison. Furthermore, novel machine algorithms were incorporated for training and testing, yielding promising outcomes. Our post-analysis of the NHANES longitudinal cohort dataset revealed an independent positive correlation between NHHR and the severity of fatty liver. NHHR demonstrates superior accuracy to conventional lipid parameters in predicting future fatty liver severity. This parameter may better represent the balance between atherogenic and anti-atherogenic lipoproteins [31], serving not only to evaluate atherosclerosis risk but also to assess the severity of fatty liver. To further investigate the value and optimal threshold of NHHR in predicting fatty liver, we constructed a machine learning predictive algorithm. The results indicated that NHHR exhibited a diagnostic efficacy with an AUC of 0.914 in predicting the severity of fatty liver disease. To the best of our knowledge, this study is the first to utilize machine learning methods to predict the impact of NHHR on the occurrence of fatty liver disease. Compared to individual lipid components, such as non-HDL-C, NHHR provides a more comprehensive reflection of the balance between atherogenic and anti-atherogenic lipoproteins. While non-HDL-C is an important indicator of lipid metabolism, it does not account for the protective role of HDL-C. In contrast, NHHR takes both factors into consideration, making it a more holistic and potentially more informative marker for assessing the risks associated with lipid metabolism disorders and their impact on MAFLD. Furthermore, although BMI is widely used as an indicator of body fat and is associated with MAFLD, it has certain limitations. BMI provides a rough estimate of body fat based solely on height and weight and does not directly measure lipid metabolism or hepatic fat accumulation. In contrast, NHHR is directly related to lipid profiles, and both our study and previous research have shown its significant correlation with the CAP, a well-established marker of hepatic fat content. This direct link between NHHR and hepatic fat makes it a more specific predictor of MAFLD. In our study, even after adjusting for multiple confounders, including BMI, NHHR remained significantly positively correlated with CAP. This finding suggests that NHHR offers additional predictive value beyond that provided by BMI.

Our study boasts several strengths. Firstly, we utilized data from a large, nationally representative sample, enhancing the generalizability of our findings to the US population. Secondly, we adjusted for various confounding factors, including demographic, lifestyle, and clinical variables, ensuring the robustness of our results. Lastly, machine learning was integrated into our analytical framework, marking a significant advancement in the predictive modeling of MAFLD. While traditional statistical methods have been effective in identifying associations between risk factors and disease outcomes, machine learning algorithms offer the advantage of handling complex, high-dimensional data, thereby enabling more nuanced predictions. Specifically, techniques such as XGBoost can uncover underlying patterns within the data that may remain undetected by conventional methods [34]. In our study, these algorithms demonstrated high accuracy in predicting the progression of MAFLD. Their inclusion represents a critical step forward in the personalized assessment of disease risk.

Despite these strengths, our study does have limitations. Firstly, although the current study population is mainly from the United States, it should be noted that even within this population, significant differences in dietary habits and lifestyles exist among different ethnic groups. These variations can potentially lead to differences in the performance of the predictive model. For example, certain ethnic groups may have a preference for specific types of food that could impact lipid metabolism and, consequently, the relationship between NHHR and MAFLD. Similarly, lifestyle factors such as physical activity levels and smoking habits, which may vary across ethnicities, can also influence the model’s effectiveness. Therefore, when generalizing the results obtained from this study, caution must be exercised, and further research involving a more diverse range of ethnic groups is essential to better understand and account for these potential differences and improve the robustness and generalizability of the predictive model. Secondly, due to the COVID-19 pandemic, NHANES surveys were suspended in March 2020, resulting in a relatively short study data duration. Thirdly, smoking is a significant confounding factor in liver disease progression and development, but due to missing smoking data in our cohort, it was not included as a covariate. Additionally, although the SHAP method helps to gain deeper insights into the importance of features in machine learning models, it only quantifies the contribution of each variable to model predictions and does not imply causality. In this study, despite the significant association found between NHHR and CAP, and NHHR being identified as an important feature in the MAFLD prediction model, this finding is based on a cross-sectional study design. Therefore, causality between NHHR and MAFLD cannot be inferred. The observed association may be influenced by unmeasured confounding factors. To establish NHHR as a biomarker for MAFLD in clinical practice, prospective studies are essential. Such studies could longitudinally track participants to observe the development of MAFLD, thus enabling a more accurate assessment of NHHR’s causal role in the disease process.

Based on the aforementioned considerations, further improvements can be made to this research. Future work should focus on enhancing the predictive performance for MAFLD, which can be achieved by increasing the training dataset, adjusting algorithm parameters, or incorporating deep learning techniques to optimize the existing model. Moreover, exploring additional potential features, such as biomarkers or clinical indicators reflecting liver physiology, metabolic processes, and environmental interactions, could further enhance predictive capabilities. More importantly, large-scale external validation studies across diverse populations are urgently needed to ensure the model’s generalizability and robustness. These steps will provide more reliable evidence for the early diagnosis and precise intervention of MAFLD.

## 5 Conclusion

Through analysis of baseline characteristics and regression outcomes, a significant positive correlation was observed between NHHR and CAP, underscoring the potential predictive efficacy of NHHR in MAFLD. The XGBoost predictive model developed incorporating NHHR demonstrates promising potential for the early detection and intervention of MAFLD. Further research is warranted to validate these findings in diverse populations and to address the limitations of our study.

## Supporting Information

S1 Fig SHAP values of variables in a validation set with a sample size of 200. (TIF)

S2 Fig Feature importance of variables in the validation set with a sample size of 200. (TIF)

S3 Fig SHAP values of variables in a validation set with a sample size of 500. (TIF)

S4 Fig Feature importance of variables in the validation set with a sample size of 500. (TIF)

S5 Fig SHAP values of variables in a validation set with a sample size of 1000. (TIF)

S6 Fig Feature importance of variables in the validation set with a sample size of 1000. (TIF)

S1 Table Information of the included study population.
(CSV)

S2 Table Data of the validation set with a sample size of 200.
(CSV)

S3 Table Data of the validation set with a sample size of 500.
(CSV)

S4 Table Data of the validation set with a sample size of 1000.
(CSV)

## References

[1] Younossi Z, Anstee QM, Marietti M, Hardy T, Henry L, Eslam M, et al. Global burden of NAFLD and NASH: trends, predictions, risk factors and prevention. Nat Rev Gastroenterol Hepatol. 2018;15(1):11–20. doi: 10.1038/nrgastro.2017.109 28930295

[2] Friedman SL, Neuschwander-Tetri BA, Rinella M, Sanyal AJ. Mechanisms of NAFLD development and therapeutic strategies. Nat Med. 2018;24(7):908–22. doi: 10.1038/s41591-018-0104-9 29967350 PMC6553468

[3] Byrne CD, Targher G. NAFLD: a multisystem disease. J Hepatol. 2015;62(1 Suppl):S47-64. doi: 10.1016/j.jhep.2014.12.012 25920090

[4] Quispe R, Martin SS, Michos ED, Lamba I, Blumenthal RS, Saeed A, et al. Remnant cholesterol predicts cardiovascular disease beyond LDL and ApoB: a primary prevention study. Eur Heart J. 2021;42(42):4324–32. doi: 10.1093/eurheartj/ehab432 34293083 PMC8572557

[5] Brunzell JD, Davidson M, Furberg CD, Goldberg RB, Howard BV, Stein JH, et al. Lipoprotein management in patients with cardiometabolic risk: consensus conference report from the American Diabetes Association and the American College of Cardiology Foundation. J Am Coll Cardiol. 2008;51(15):1512–24. doi: 10.1016/j.jacc.2008.02.034 18402913

[6] Orakzai SH, Nasir K, Blaha M, Blumenthal RS, Raggi P. Non-HDL cholesterol is strongly associated with coronary artery calcification in asymptomatic individuals. Atherosclerosis. 2009;202(1):289–95. doi: 10.1016/j.atherosclerosis.2008.03.014 18452924

[7] Xie J, Huang H, Liu Z, Li Y, Yu C, Xu L, et al. The associations between modifiable risk factors and nonalcoholic fatty liver disease: a comprehensive Mendelian randomization study. Hepatology. 2023;77(3):949–64. doi: 10.1002/hep.32728 35971878

[8] Yuan S, Chen J, Li X, Fan R, Arsenault B, Gill D, et al. Lifestyle and metabolic factors for nonalcoholic fatty liver disease: Mendelian randomization study. Eur J Epidemiol. 2022;37(7):723–33. doi: 10.1007/s10654-022-00868-3 35488966 PMC9329390

[9] Guo X, Yin X, Liu Z, Wang J. Non-Alcoholic Fatty Liver Disease (NAFLD) pathogenesis and natural products for prevention and treatment. Int J Mol Sci. 2022;23(24). 10.3390/ijms232415489 PMC9779435 36555127

[10] Cobbina E, Akhlaghi F. Non-alcoholic fatty liver disease (NAFLD) - pathogenesis, classification, and effect on drug metabolizing enzymes and transporters. Drug Metab Rev. 2017;49(2):197–211. doi: 10.1080/03602532.2017.1293683 28303724 PMC5576152

[11] Johnson C, Dohrmann S, Burt V, Mohadjer L. National health and nutrition examination survey: sample design, 2011-2014. Vital Health Stat 2. 2014;2014(162):1–33. 25569458

[12] Luo N, Zhang X, Huang J, Chen H, Tang H. Prevalence of steatotic liver disease and associated fibrosis in the United States: Results from NHANES 2017-March 2020. J Hepatol. 2024;80(2):e70–1. doi: 10.1016/j.jhep.2023.08.019 37647990

[13] Hou K, Song W, He J, Ma Z. The association between non-high-density lipoprotein cholesterol to high-density lipoprotein cholesterol ratio (NHHR) and prevalence of periodontitis among US adults: a cross-sectional NHANES study. Sci Rep. 2024;14(1):5558. doi: 10.1038/s41598-024-56276-y 38448487 PMC10918089

[14] Liu C, Chien L. Predictive role of neutrophil-percentage-to-albumin ratio (npar) in nonalcoholic fatty liver disease and advanced liver fibrosis in nondiabetic us adults: Evidence from nhanes 2017-2018. Nutrients. 2023;15(8). 10.3390/nu15081892 PMC10141547 37111111

[15] Ginès P, Krag A, Abraldes JG, Solà E, Fabrellas N, Kamath PS. Liver cirrhosis. Lancet. 2021;398(10308):1359–76. doi: 10.1016/S0140-6736(21)01374-X 34543610

[16] Castera L, Cusi K. Diabetes and cirrhosis: current concepts on diagnosis and management. Hepatology. 2023;77(6):2128–46. doi: 10.1097/HEP.0000000000000263 36631005

[17] Eslam M, Newsome PN, Sarin SK, Anstee QM, Targher G, Romero-Gomez M, et al. A new definition for metabolic dysfunction-associated fatty liver disease: An international expert consensus statement. J Hepatol. 2020;73(1):202–9. doi: 10.1016/j.jhep.2020.03.039 32278004

[18] Sasso M, Beaugrand M, de Ledinghen V, Douvin C, Marcellin P, Poupon R, et al. Controlled attenuation parameter (CAP): a novel VCTE™ guided ultrasonic attenuation measurement for the evaluation of hepatic steatosis: preliminary study and validation in a cohort of patients with chronic liver disease from various causes. Ultrasound Med Biol. 2010;36(11):1825–35. doi: 10.1016/j.ultrasmedbio.2010.07.005 20870345

[19] Yen Y-H, Chen J-B, Cheng B-C, Chen J-F, Chang K-C, Tseng P-L, et al. Using controlled attenuation parameter combined with ultrasound to survey non-alcoholic fatty liver disease in hemodialysis patients: A prospective cohort study. PLoS One. 2017;12(4):e0176027. doi: 10.1371/journal.pone.0176027 28426815 PMC5398606

[20] Huang Z, Ng K, Chen H, Deng W, Li Y. Validation of controlled attenuation parameter measured by fibroscan as a novel surrogate marker for the evaluation of metabolic derangement. Front Endocrinol (Lausanne). 2022;12:739875. doi: 10.3389/fendo.2021.739875 35173677 PMC8841525

[21] Mikolasevic I, Orlic L, Zaputovic L, Racki S, Cubranic Z, Anic K, et al. Usefulness of liver test and controlled attenuation parameter in detection of nonalcoholic fatty liver disease in patients with chronic renal failure and coronary heart disease. Wien Klin Wochenschr. 2015;127(11–12):451–8. doi: 10.1007/s00508-015-0757-z 25854911

[22] Wang K, Zhao Y, Nie J, Xu H, Yu C, Wang S. Higher HEI-2015 score is associated with reduced risk of depression: result from NHANES 2005-2016. Nutrients. 2021;13(2). 10.3390/nu13020348 PMC7911826 33503826

[23] Corey KE, Lai M, Gelrud LG, Misdraji J, Barlow LL, Zheng H, et al. Non-high-density lipoprotein cholesterol as a biomarker for nonalcoholic steatohepatitis. Clin Gastroenterol Hepatol. 2012;10(6):651–6. doi: 10.1016/j.cgh.2012.01.017 22330232 PMC3360815

[24] Alkhouri N, Eng K, Lopez R, Nobili V. Non-high-density lipoprotein cholesterol (non-HDL-C) levels in children with nonalcoholic fatty liver disease (NAFLD). Springerplus. 2014;3:407. doi: 10.1186/2193-1801-3-407 25126490 PMC4130966

[25] Wang D, Wang L, Wang Z, Chen S, Ni Y, Jiang D. Higher non-HDL-cholesterol to HDL-cholesterol ratio linked with increased nonalcoholic steatohepatitis. Lipids Health Dis. 2018;17(1):67. doi: 10.1186/s12944-018-0720-x 29615042 PMC5883308

[26] Gao S, Ramen K, Yu S, Luo J. Higher non-HDL-cholesterol to HDL-cholesterol ratio is linked to increase in non-alcoholic fatty liver disease: secondary analysis based on a longitudinal study. Int J Clin Exp Pathol. 2020;13(10):2569–75. 33165444 PMC7642706

[27] Yang S, Zhong J, Ye M, Miao L, Lu G, Xu C, et al. Association between the non-HDL-cholesterol to HDL-cholesterol ratio and non-alcoholic fatty liver disease in Chinese children and adolescents: a large single-center cross-sectional study. Lipids Health Dis. 2020;19(1):242. doi: 10.1186/s12944-020-01421-5 33222696 PMC7681973

[28] Qing G, Deng W, Zhou Y, Zheng L, Wang Y, Wei B. The association between non-high-density lipoprotein cholesterol to high-density lipoprotein cholesterol ratio (NHHR) and suicidal ideation in adults: a population-based study in the United States. Lipids Health Dis. 2024;23(1):17. doi: 10.1186/s12944-024-02012-4 38218917 PMC10788025

[29] Qi X, Wang S, Huang Q, Chen X, Qiu L, Ouyang K, et al. The association between non-high-density lipoprotein cholesterol to high-density lipoprotein cholesterol ratio (NHHR) and risk of depression among US adults: A cross-sectional NHANES study. J Affect Disord. 2024;344:451–7. doi: 10.1016/j.jad.2023.10.064 37838268

[30] Hou K, Song W, He J, Ma Z. The association between non-high-density lipoprotein cholesterol to high-density lipoprotein cholesterol ratio (NHHR) and prevalence of periodontitis among US adults: a cross-sectional NHANES study. Sci. Rep. 2024;14(1):5558. doi: insert_doi_here 38448487 10.1038/s41598-024-56276-y PMC10918089

[31] Sheng G, Liu D, Kuang M, Zhong Y, Zhang S, Zou Y. Utility of non-high-density lipoprotein cholesterol to high-density lipoprotein cholesterol ratio in evaluating incident diabetes risk. Diabetes Metab Syndr Obes. 2022;15:1677–86. doi: 10.2147/DMSO.S355980 35669362 PMC9166911

[32] Lin W, Luo S, Li W, Liu J, Zhou T, Yang F, et al. Association between the non-HDL-cholesterol to HDL- cholesterol ratio and abdominal aortic aneurysm from a Chinese screening program. Lipids Health Dis. 2023;22(1):187. doi: 10.1186/s12944-023-01939-4 37932803 PMC10626699

[33] Butt AA, Yan P, Simon TG, Chung RT, Abou-Samra A-B; ERCHIVES study team. Changes in circulating lipids level over time after acquiring HCV infection: results from ERCHIVES. BMC Infect Dis. 2015;15:510. doi: 10.1186/s12879-015-1268-2 26558512 PMC4642733

[34] Chen T, Guestrin C, ^editors. XGBoost: A Scalable Tree Boosting System; 2016 2016/1/1; New York, NY, USA. Pub Place: ACM; Year Published.

