# Supplementary figures and images for: Machine learning for predicting metabolic-associated fatty liver disease including NHHR: a cross-sectional NHANES study
Source: PLoS One. 2025 Mar 18;20(3):e0319851. doi: 10.1371/journal.pone.0319851 (PMC11918377; doi:10.1371/journal.pone.0319851)

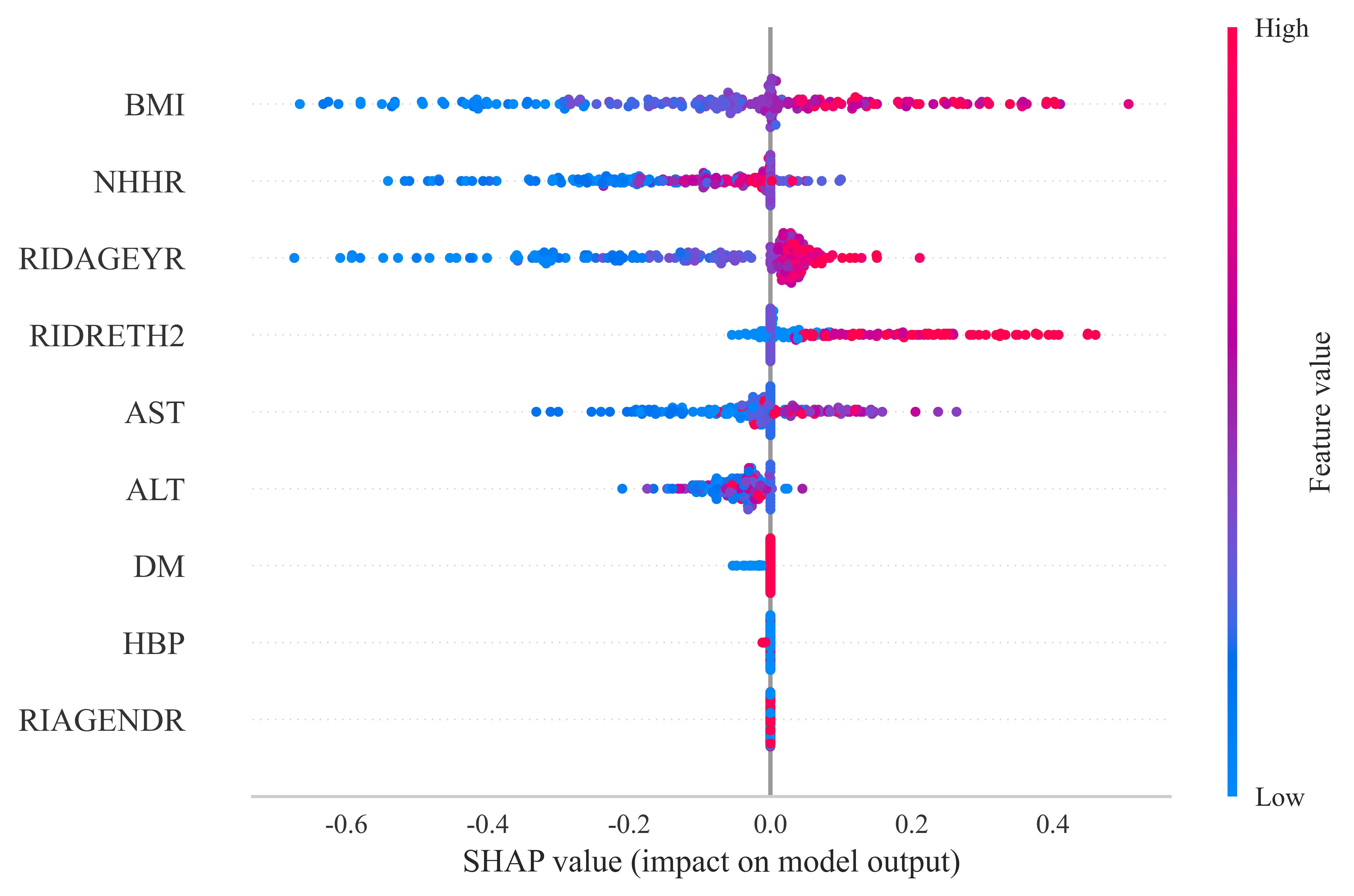

Supplement: S1 Fig — (TIF) [file pone.0319851.s001.tif]

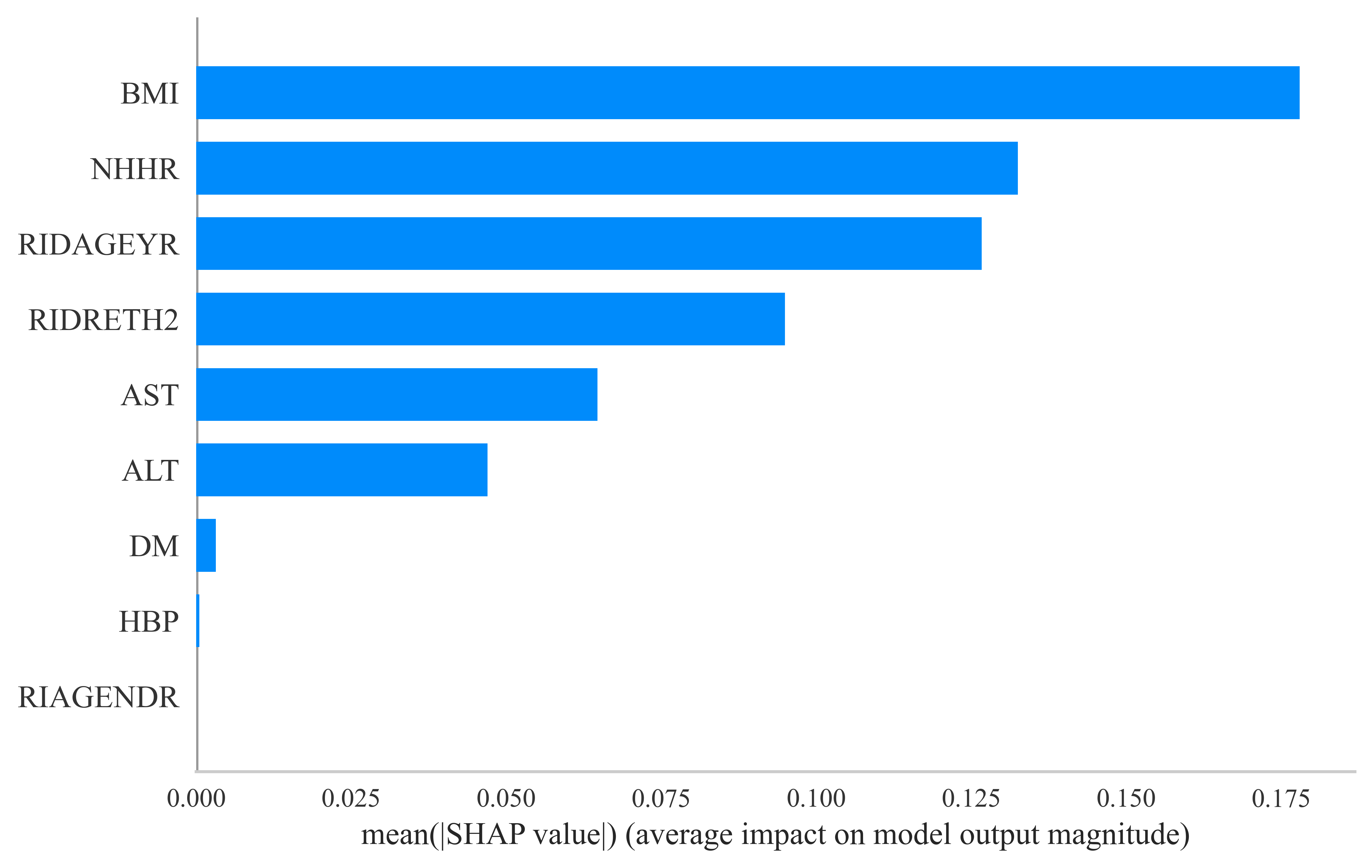

Supplement: S2 Fig — (TIF) [file pone.0319851.s002.tif]

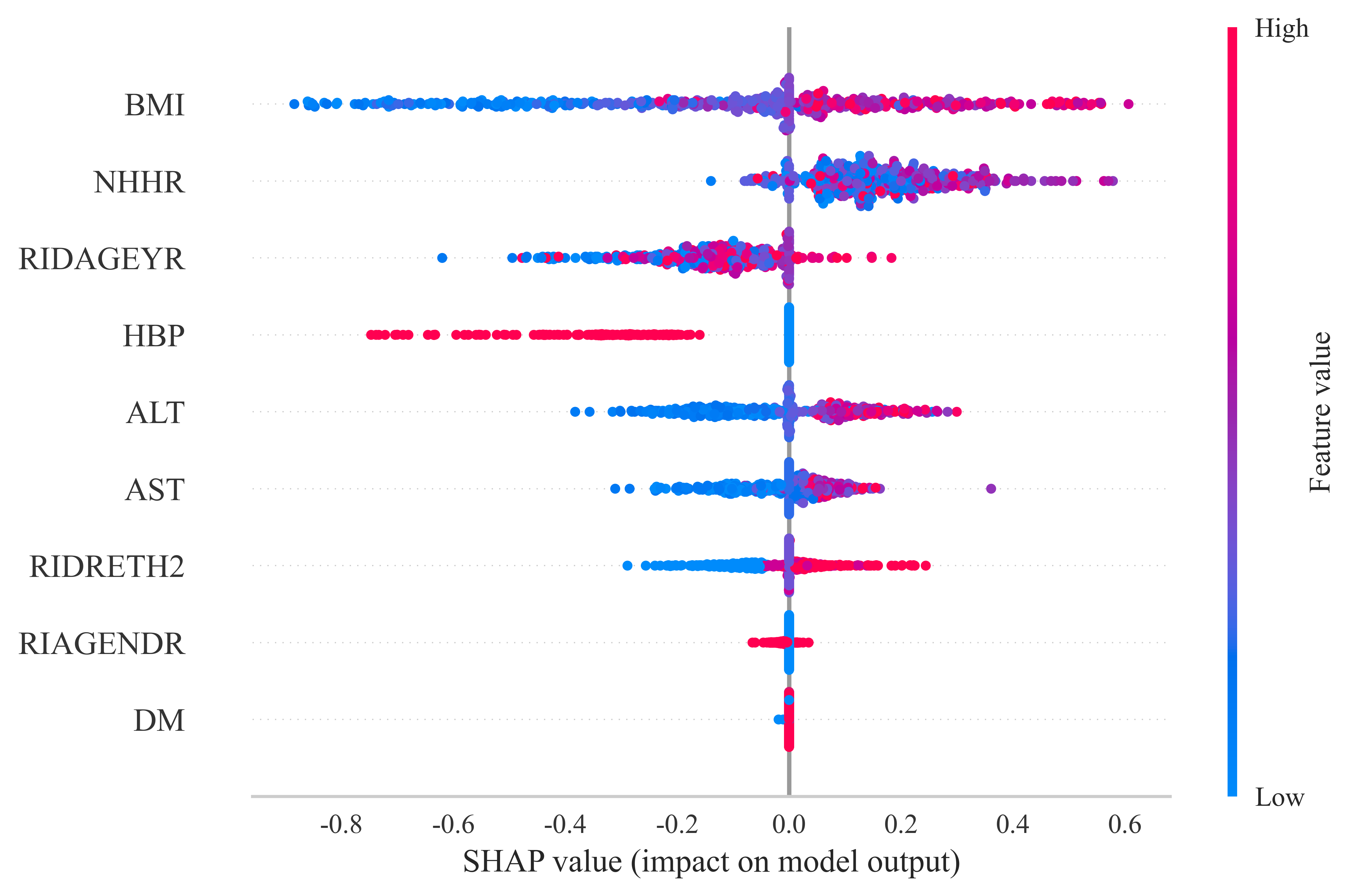

Supplement: S3 Fig — (TIF) [file pone.0319851.s003.tif]

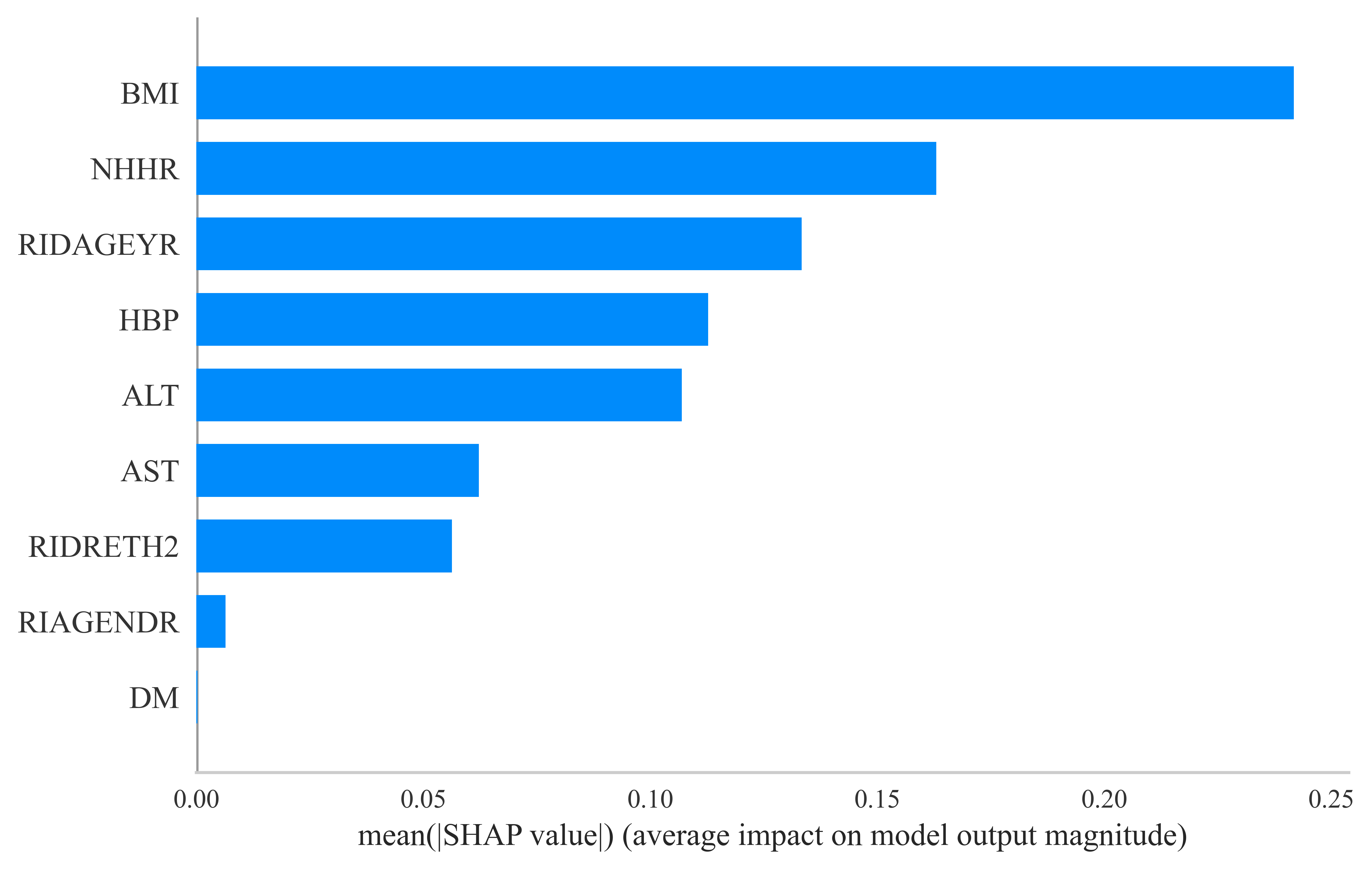

Supplement: S4 Fig — (TIF) [file pone.0319851.s004.tif]

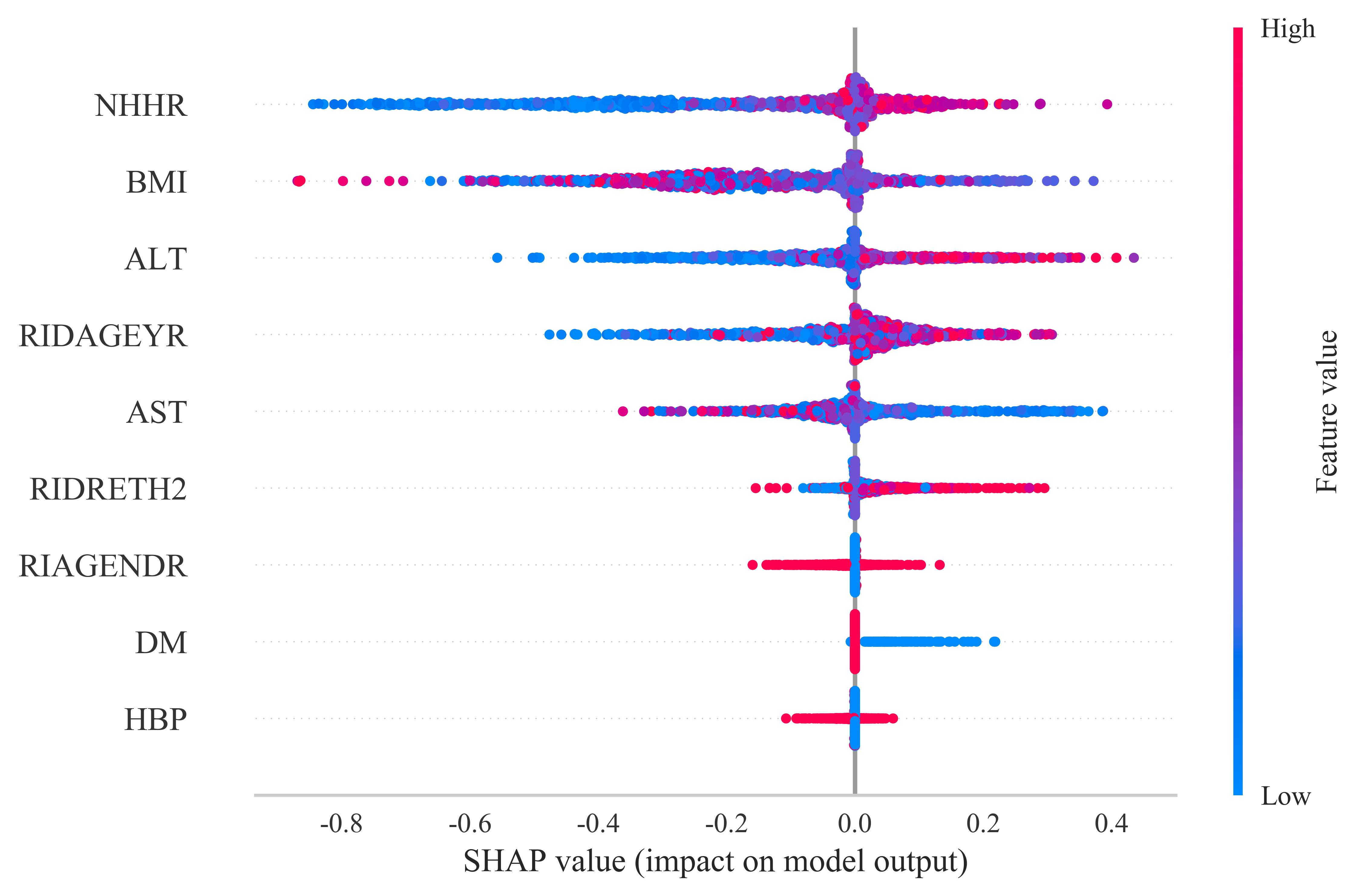

Supplement: S5 Fig — (TIF) [file pone.0319851.s005.tif]

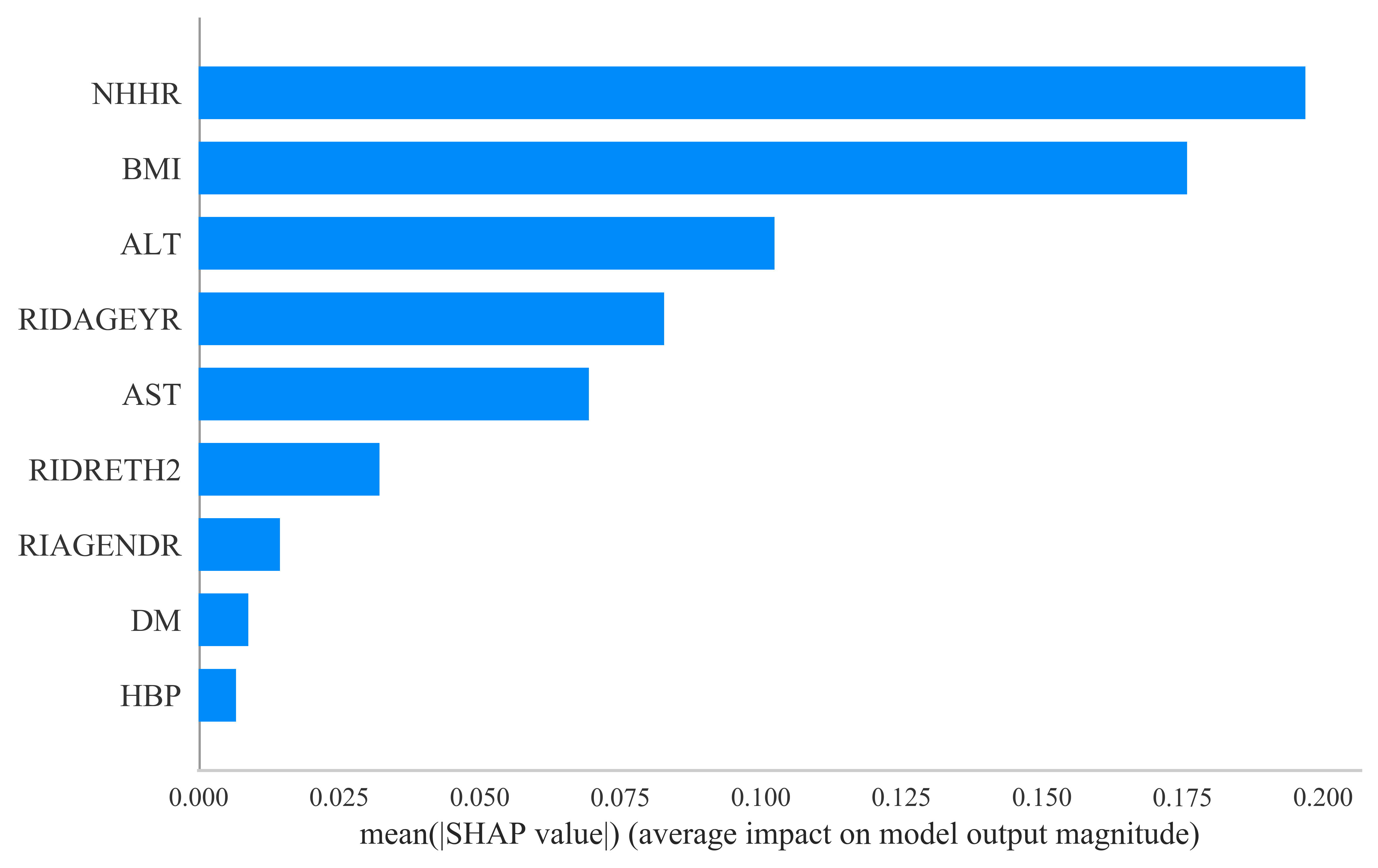

Supplement: S6 Fig — (TIF) [file pone.0319851.s006.tif]
